# Functionally rich crop rotations increase calorie and macronutrient outputs across Europe

**DOI:** 10.1038/s43016-026-01293-5

**Published:** 2026-02-25

**Authors:** Giulia Vico, Alessio Costa, Monique E. Smith, Timothy Bowles, Amélie C. M. Gaudin, Christine A. Watson, Guido Baldoni, Antonio Berti, Andrzej Blecharczyk, Krzysztof Jonczyk, Martina Mazzon, Claudio Marzadori, Francesco Morari, Lorenzo Negri, Andrea Onofri, José Luis Tenorio Pasamón, Boël Sandström, Inés Santín-Montanyá, Zuzanna Sawinska, Jarosław Stalenga, Francesco Tei, Cairistiona F. E. Topp, Robin L. Walker, Riccardo Bommarco

**Affiliations:** 1 Department of Ecology, Swedish University of Agricultural Sciences, Uppsala, Sweden; 2 Department of Crop Production Ecology, Swedish University of Agricultural Sciences, Uppsala, Sweden; 3 Department of Environmental Science, Policy and Management, University of California Berkeley, Berkeley, CA USA; 4 Department of Plant Sciences, University of California Davis, Davis, CA USA; 5 Scotland’s Rural College, Aberdeen, UK; 6 Department of Agricultural and Food Sciences, University of Bologna, Bologna, Italy; 7 Department of Agronomy, Food, Natural Resources, Animals and Environment, University of Padova, Padova, Italy; 8 Department of Agronomy, Poznań University of Life Sciences, Poznań, Poland; 9 Department of Agroecology and Economics, Institute of Soil Science and Plant Cultivation—State Research Institute, Puławy, Poland; 10 Department of Agricultural Food and Environmental Sciences, University of Perugia, Perugia, Italy; 11 Environment and Agronomy Department, Instituto Nacional de Investigación y Tecnología Agraria y Alimentaria (INIA-CSIC), Madrid, Spain; 12 Department of Crop Production Ecology, Swedish University of Agricultural Sciences, Umeå, Sweden; 13 Scotland’s Rural College, Edinburgh, UK

**Keywords:** Agriculture, Agroecology

## Abstract

Increased crop diversity in cereal-dominated rotations can enhance crop protection, nutrient use efficiency and climate change adaptation. Nevertheless, it is argued that replacing cereals in rotations diminishes food production, threatening food security. Here we compared outputs of calories and macronutrients (carbohydrates, proteins, fats) for human consumption from cereal monocultures, cereal-only rotations and rotations including two or three functionally distinct crop types (cereals plus root and oil crops, legumes or ley) in 16 long-term experiments across Europe. Rotations with three functional types produced more calories and macronutrients than cereal monocultures and cereal-only rotations with forage crops used to produce milk. Carbohydrate gains depended on growing conditions and crop choice. Advantages increased over time but were lost with forage crops used for beef or biofuel. Functionally rich rotations provided macronutrient proportions closer to recommended human diets. Our analysis shows no trade-off between functionally rich rotations and food production or agricultural land expansion.

## Abstract (web summary)

While the benefits of crop diversity are known, doubts remain as to whether replacing cereals in rotations reduces nutrient production. A comparison of 16 long-term field experiments across Europe shows no trade-off between functionally rich rotations and food productivity.

## Main

In industrial agriculture, diverse cropping systems have been replaced by monocultures and short crop rotations^1–5^. These simplified rotations rely heavily on synthetic inputs of mineral fertilizers and pesticides, causing negative effects on soil quality, climate and the environment^6^. Alternative approaches are needed to provide sufficient and nutritious food, and to reduce the environmental impacts of crop production^7,8^.

Evidence is mounting that diverse crop rotations can maintain or increase yields of staple cereals with reduced negative environmental effects and vulnerability to climate change^9–12^. Nevertheless, food security is often brought in as a counterargument to diverse rotations, because other, purportedly less productive, crops would replace the cereals. Producing enough food for a growing population is a cornerstone argument for establishing specialized input-intensive cereal cropping across vast regions^13^. Furthermore, comparative advantages in the transnational food trade are assumed to be achieved by concentrating production of a specific cereal to the part of the world where it grows particularly well, such as maize in the US Midwest or wheat in central and northern Europe. These ‘feed the world’ arguments focus on caloric sufficiency and the role of cereals in producing abundant carbohydrates. Four staple crops, which include three cereals, were claimed to produce more calories, oil and protein per unit cultivated land than many other crops, from which it was concluded that diversified rotations would require expanded agricultural land to satisfy growing food demands^14^. However, the feasibility of more diverse crop rotations needs to be evaluated based on the output of macronutrients from all of the crops in the rotation.

Functional richness (FR) indicates the diversity in crop types in a rotation, such as cereals, annual legumes, broadleaves and ley. Leys are generally mixtures of, or monocrops of, perennial grasses and/or legumes for feed. Functional crop types have distinct calorie and macronutrient contents and ecological niches, thereby complementing each other for pest regulation and resource use. Long-term experiments show that cereal yields and nitrogen use efficiency were higher and increased over time when the cereal was part of a functionally rich rotation^9–11^. However, the output from the entire rotation has rarely been considered^15–17^ and it remains unclear whether functionally rich rotations provide more or fewer calories and macronutrients compared with a mono-sequence of one cereal species, or a cereal-only rotation. In a North American long-term experiment spanning 26 years, whole-rotation calorie output was 23–30% lower in diverse rotations compared with maize monoculture^16^. However, analysing only calories hides the potential of diverse rotations to provide carbohydrates, proteins and fats, and to do so in proportions closer to those required for healthy human diets compared with crops dominated by cereals^18^. Comparing calorie and macronutrient outputs between cereal-dominated and functionally rich rotations would allow for a more comprehensive assessment of whether crop diversity can promote food security and nutritionally balanced diets.

The rotational output for human nutrition depends heavily on whether the crops are consumed directly as human food or not. Some crops, such as ley, cannot be eaten directly by humans and are used to produce dairy, meat or biofuel. Even crops that are suitable for direct human consumption are often used to produce animal products or biofuel^19,20^. The conversion efficiency into human food depends on the final product and is generally low for feed^19–21^ and none for biofuel. It remains to be examined how FR affects calorie or macronutrient outputs depending on the use of crops that cannot be consumed directly by humans.

**Extended Data Fig. 1:**
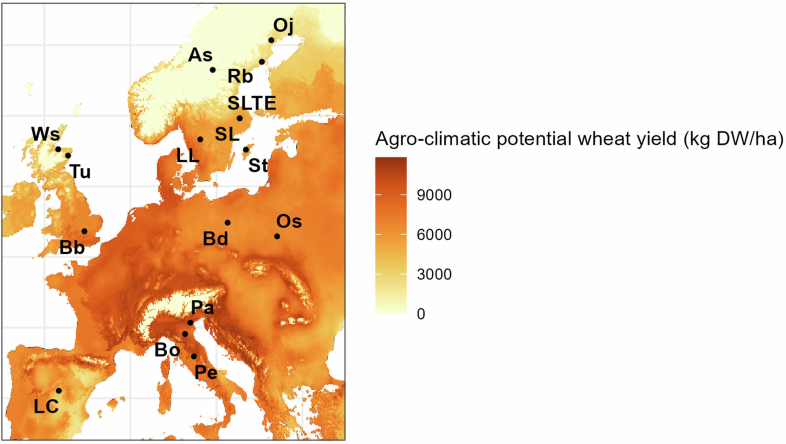
Location of the long-term agricultural experiments. See Supplementary Table 1 for the clarification of site codes and additional site information. The background map is the agro-climatic potential yield of wheat extracted from the GAEZv4 database (Fischer, G. Global Agro-Ecological Zones v4 – Model Documentation. FAO, 2021), available at https://gaez.fao.org/pages/data-viewer (sourced in June 2025).

We combined crop yield data from 16 representative long-term (11–53 years) experiments across Europe, with a total of >34,500 yield observations (Extended Data Fig. 1 and Supplementary Table 1), and nutritional values of commonly used retail products obtained from each crop (Supplementary Information Table 2). We explored the effect of FR on both calorie and macronutrient (carbohydrate, protein and fat) outputs of entire rotations. The functional crop types included in our analyses were cereals, annual legumes, broadleaves (that is, oil and root crops) and ley. We also considered multiple end uses for cereals, broadleaves, legumes and ley not directly usable for human consumption (hereafter, forage crops). Among the cereal-dominated rotations, we separated monocultures, which grew the same cereal species year after year (FR 1M), from cereal-only rotations, which rotated different cereal species (FR 1C). We hypothesized that (1) calorie outputs decrease at higher FR; (2) carbohydrate outputs diminish, and protein and fat outputs increase, with increasing FR; and (3) FR enhances both calorie and macronutrient outputs over time. We further hypothesized that carbohydrate outputs decrease more with FR if forage crops were used to produce beef and biofuel instead of milk.

**Table 1:** Effect of functional richness (FR) and time from rotation implementation on calorie and macronutrient outputs, when forage is used for milk production

Coefficients—forage for milk (equation (3))	Predictors	Units	(1) Calorie (Gcal ha^−1^ yr^−1^)			(2) Carbohydrate (kg ha^−1^ yr^−1^)			(3) Protein (kg ha^−1^ yr^−1^)			(4) Fat (kg ha^−1^ yr^−1^)		
			Estimate	s.e.	*P*	Estimate	s.e.	*P*	Estimate	s.e.	*P*	Estimate	s.e.	*P*
${\beta }_{0}$	Intercept	Gcal ha^−1^ yr^−1^ or kg ha^−1^ yr^−1^	3.3	0.19	<0.001	47.8	2.26	<0.001	18.9	1.2	<0.001	8.8	1.4	<0.001
${\beta }_{t}$	Time	Gcal ha^−1^ yr^−2^ or kg ha^−1^ yr^−2^	−8.2	2.38	<0.001	−156.0	32.0	<0.001	−43.0	14.2	0.003	19.9	12.9	0.125
${\beta }_{{t}^{2}}$	Time^2^	Gcal ha^−1^ yr^−3^ or kg ha^−1^ yr^−3^	10.7	2.40	<0.001	125.0	32.7	<0.001	67.3	14.2	<0.001	64.8	13.3	<0.001
${\beta }_{{\mathrm{FR}}_{1{\rm{C}}}}$	FR 1C	Gcal ha^−1^ yr^−1^ or kg ha^−1^ yr^−1^	−0.29	0.02	<0.001	−4.92	0.27	<0.001	−2.3	0.10	<0.001	0.45	0.11	<0.001
${\beta }_{{\mathrm{FR}}_{2}}$	FR 2	Gcal ha^−1^ yr^−1^ or kg ha^−1^ yr^−1^	−0.49	0.02	<0.001	−12.3	0.30	<0.001	−1.4	0.11	<0.001	4.4	0.12	<0.001
${\beta }_{{\mathrm{FR}}_{3}}$	FR 3	Gcal ha^−1^ yr^−1^ or kg ha^−1^ yr^−1^	1.7	0.03	<0.001	5.36	0.35	<0.001	14.5	0.14	<0.001	23.6	0.15	<0.001
${\beta }_{{\mathrm{FR}}_{1{\rm{C}}}t}$	Time:FR 1C	Gcal ha^−1^ yr^−2^ or kg ha^−1^ yr^−2^	15.0	2.31	<0.001	282.0	32.4	<0.001	89.8	13.0	<0.001	−0.07	13.9	0.996
${\beta }_{{\mathrm{FR}}_{1{\rm{C}}}{t}^{2}}$	Time^2^:FR 1C	Gcal ha^−1^ yr^−3^ or kg ha^−1^ yr^−3^	−14.5	2.44	<0.001	−227.0	34.2	<0.001	−65.3	13.7	<0.001	−27.1	14.7	0.064
${\beta }_{{\mathrm{FR}}_{2}t}$	Time:FR 2	Gcal ha^−1^ yr^−2^ or kg ha^−1^ yr^−2^	2.68	2.27	0.238	126.0	32.0	<0.001	−20.1	12.8	0.116	−67.8	13.7	<0.001
${\beta }_{{\mathrm{FR}}_{2}{t}^{2}}$	Time^2^:FR 2	Gcal ha^−1^ yr^−3^ or kg ha^−1^ yr^−3^	−0.31	2.40	0.897	16.1	33.7	0.634	−15.3	13.6	0.259	−22.3	14.4	0.121
${\beta }_{{\mathrm{FR}}_{3}t}$	Time:FR 3	Gcal ha^−1^ yr^−2^ or kg ha^−1^ yr^−2^	16.8	2.69	<0.001	136.0	37.6	<0.001	125.0	15.3	<0.001	97.3	15.9	<0.001
${\beta }_{{\mathrm{FR}}_{3}{t}^{2}}$	Time^2^:FR 3	Gcal ha^−1^ yr^−3^ or kg ha^−1^ yr^−3^	−23.5	2.61	<0.001	−231.0	36.5	<0.001	−161.0	14.9	<0.001	−164.0	15.4	<0.001
Marginal *R*^2^			0.28			0.13			0.41			0.56		
Conditional *R*^2^			0.72			0.61			0.80			0.84		

Fitted model parameters for the square-rooted annual outputs (equation (3)): (1) calories (Gcal ha^−1^ yr^−1^), (2) carbohydrates (kg ha^−1^ yr^−1^), (3) proteins (kg ha^−1^ yr^−1^) and (4) fats (kg ha^−1^ yr^−1^). The intercept is the output of cereal monocultures at time 0 (FR 1M). The two-sided *P* values were determined using Satterthwaite’s approximation to calculate the degrees of freedom. The marginal *R*^2^ is the variation explained by the fixed factors alone, while the conditional *R*^2^ is the variation explained by the entire model including fixed and random factors.

## Results

### Outputs with forage crops used for milk production

**Fig. 1:**
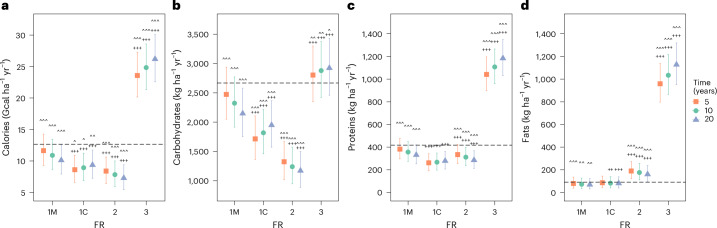
Model-predicted effects of functional richness (FR) on annual crop rotation outputs when forage crops are used for milk production. **a**–**d**, Calories (**a**) and macronutrients (carbohydrates (**b**), proteins (**c**) and fats (**d**)) produced per year by all the crops in the rotation, when they cover 1 ha. FR is the number of functional types included in the rotation, with FR 1M referring to a cereal monoculture and FR 1C to a cereal-only rotation. The statistical models were obtained by fitting the 12,517 whole-rotation outputs relative to the 16 long-term experiments (see Supplementary Table 1 for details). Horizontal dashed lines are model predictions relative to FR 1M at time 0. Filled symbols refer to model predictions after 5 years (orange squares), 10 years (green circles) and 20 years (blue triangles) following the implementation of the rotation—times arbitrarily chosen to show changes over time. Whiskers extend over the 5–95% confidence intervals. Symbols indicating significances refer to contrasts across levels of FR within each time, using FR 1M at the same time as the baseline (+, *P* < 0.05; ++, *P* < 0.01; +++, *P* < 0.001), and across time within each FR with time 0 at that same FR as the baseline (^, *P* < 0.05; ^^, *P* < 0.01; ^^^, *P* < 0.001). Post hoc two-sided tests were adjusted for multiplicity using multivariate *t*-distribution. Note the difference in the *y* axis scale among plots.

Functionally diverse rotations with three crop types (FR 3) increased calorie and macronutrient outputs compared with cereal monocultures (FR 1M), when assuming that forage crops were used for milk production (Table 1 and Fig. 1: contrasts indicated by + symbols). These effects were already present 5 years after the start of the long-term experiments. Compared with the benchmark of cereal monoculture (FR 1M) at year 0, FR 3 5 years after implementation produced 85% more calories, that is, 23.6 Gcal ha^−1^ yr^−1^ (5–95% confidence interval (CI) [20.2, 27.2]) instead of 12.7 Gcal ha^−1^ yr^−1^ (5–95% CI [10.2, 15.4]). Using the same benchmark, protein output was more than doubled, that is, 1,041 kg ha^−1^ yr^−1^ (95% CI [897, 1,196]) instead of 416 kg ha^−1^ yr^−1^ (5–95% CI [327, 520]) and fats were tenfold higher, that is, 959 kg ha^−1^ yr^−1^ (95% CI [796, 1,137]) compared with 91 kg ha^−1^ yr^−1^ (5–95% CI [46, 151]). Carbohydrate outputs of FR 3 5 years after implementation were 5% higher than those of FR 1M at year 0, that is, 2,803 kg ha^−1^ yr^−1^ (95% CI [2,348, 3,298]) instead of 2,663 kg ha^−1^ yr^−1^ (5–95% CI [2,218, 3,158]). However, using the same term of comparison, carbohydrate outputs of FR 3 were 9% lower when not considering the data from one site—Padova (Supplementary Fig. 3)—pointing to the importance of pedoclimatic conditions and rotated crops to define whether carbohydrates benefit from diverse rotations.

Functional diversity also increased the outputs over time (Table 1 and Fig. 1; contrasts indicated by ^ symbols). Specifically, over the 15 years spanning 5–20 years after implementation, calorie output increased in FR 3 by 11% to 26.2 Gcal ha^−1^ yr^−1^ (5–95% CI [22.6, 30.0]). Over the same period, carbohydrate outputs of FR 3 increased by 4% to 2,922 kg ha^−1^ yr^−1^ (5–95% CI [2,458, 3,427]), proteins by 14% to 1,184 kg ha^−1^ yr^−1^ (5–95% CI [1,030, 1,348]) and fats by 18% to 1,128 kg ha^−1^ yr^−1^ (5–95% CI [951, 1,320]). The increases in carbohydrate outputs at FR 3 and decline in FR 1M with time led to a benefit in carbohydrates from FR 3 of 36% 20 years after implementation compared with FR 1M at the same time. However, carbohydrate outputs did not change in time in both FR 1M and FR 3 when the Padova site was not considered (Supplementary Fig. 3).

Rotations including a single functional type beyond cereals (FR 2) produced 28% fewer calories, 46% fewer carbohydrates and 13% fewer proteins 5 years after implementation, compared with FR 1M at the same time (Table 1 and Fig. 1; contrasts indicated by + symbols). However, FR 2 led to 139% and 115% higher fat outputs compared with 1M after 5 years and 10 years since implementation, respectively. Fat outputs of FR 2 decreased over time (Fig. 1; contrasts indicated by ^ symbols).

Having multiple cereals in a rotation (FR 1C) gave lower outputs of all kinds except for fats after 5 years since implementation, compared with FR 1M at the same time (Fig. 1; contrasts indicated by + symbols), except carbohydrates if removing long-term experiment Broadbalk (Supplementary Fig. 2). Calories and carbohydrates increased over time for FR 1C, whereas all outputs decreased over time for FR 1M (Fig. 1; contrasts indicated by ^ symbols). However, removing the Padova site led to no change in time at FR 1M, except for proteins.

### Outputs depending on the use of forage crops

**Fig. 2:**
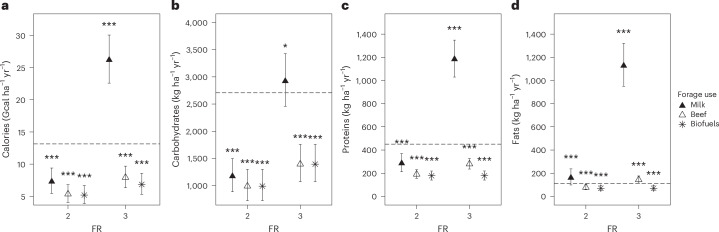
Effect of forage crop use (milk, beef or biofuel production) on outputs at contrasting levels of functional richness (FR). **a**–**d**, Model estimates 20 years after implementation for calorie (**a**), carbohydrate (**b**), protein (**c**) and fat (**d**) outputs. Symbols are model predictions when forage is used to produce milk (filled triangles), beef (open triangles) and biofuel (stars; Table 1 and Extended Data Tables 1 and 2). FR is the number of functional types included in the rotation, with FR 1M referring to a cereal monoculture and FR 1C a cereal-only rotation. The statistical models were obtained by fitting the 12,517 whole-rotation outputs relative to the 16 long-term experiments (Supplementary Table 1). Whiskers extend over the 5–95% confidence intervals. Horizontal dashed lines mark model-predicted outputs for the cereal monoculture (FR 1M) at year 0. Output changes across forage crop use of cereal monocultures and cereal-only rotations are not reported, as they do not include any forage crops (Supplementary Table 1). Significances refer to contrasts between each FR and forage crop use with FR 1M and time 0 as baseline (*P < 0.05; **P < 0.01; ***P < 0.001). Post hoc two-sided tests were adjusted for multiplicity using multivariate *t*-distribution.

**Extended Data Fig. 2:**
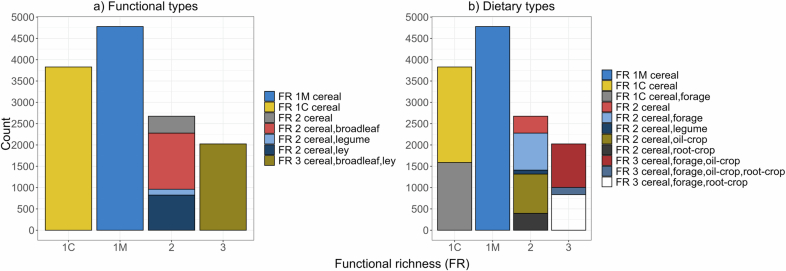
Number of observations of whole rotation macronutrient outputs, grouped according to functional richness FR, that is, the number of functional types included in the rotation. Colours represent observations from rotations that include combinations of (**a**) the functional types considered, separating cereal monocultures (FR 1M) from cereal-only rotations (FR 1C) and (**b**) five distinct dietary types. In **b**), cereal, legume, oil crop and root crop include all cereals, legumes, oil crops, and root crops intended for direct human consumption, respectively. Forage include ley, cereals, legumes, oil and root crops intended for feed. The rotations were selected so that all monocultures (FR 1M) are of cereals.

Converting forage crops to beef or biofuel, instead of milk, negated the benefits of FR 3, resulting in lower calorie, carbohydrate and protein outputs compared with FR 1M at time 0 (Fig. 2 and Extended Data Tables 1 and 2). Only fats were higher when forage crops were used for beef production in FR 3 (Fig. 2). For FR 2, calorie and macronutrient outputs were less dependent on the end use of forage crops (Fig. 2 and Extended Data Tables 1 and 2) probably because fewer forage crops were included in rotations at this FR (Extended Data Fig. 2b).

## Discussion

We found that functionally rich crop rotations can increase the production of calories. This contradicted both our first hypothesis and widespread arguments against diverse cropping systems assumed to weaken food security or require increased agricultural land. Instead, rotating three functional types produced more calories within just 5 years following adoption compared with cereal monocultures, assuming that forage crops were used to produce milk. This result can be partially explained by 64% of the observations from rotations with three functional types pertaining to rotations including oil crops (Supplementary Table 1). Oilseeds have high caloric content per unit mass (Supplementary Table 2). Rotations including two functional types beyond cereals can thus provide more calories than cereal monocultures and cereal-only rotations without requiring additional agricultural land in pedoclimatic zones similar to our long-term experiments. However, rotating multiple cereal species resulted in lower calorie output compared with a cereal monoculture. The benefits of a higher cereal species richness were probably counteracted by combining less productive species in the rotations, such as replacing a winter-sown cereal with lower-yielding spring-sown cereals, or replacing maize with a small-grain cereal. This mechanism could also explain the decreased calorie output from rotating cereals together with a single additional functional type, which partially confirmed our first hypothesis that functional crop diversity reduces calorie outputs. A comparison between maize monoculture and rotations with two functional types in a long-term experiment in North America showed a similar result^16^.

In line with our second hypothesis, rotations including cereals together with two additional functional types produced more proteins and fats when forage was used for milk, compared with cereal monocultures and rotations with only cereals. Carbohydrate outputs were also higher than those of a cereal monoculture, but this advantage was lost when removing the data from the long-term experiment in Padova. This was a highly productive site (Extended Data Fig. 1), where the diverse rotation included 2 years of maize and one of sugar beet (Supplementary Table 1). Partially in contrast with our second hypothesis, we conclude that carbohydrate outputs can decrease or increase with diversity, depending on pedoclimatic conditions and choice of rotated crops. The carbohydrate gains occurred despite the functionally rich rotations having fewer carbohydrate- and more protein- and fat-rich crops, such as legumes and oilseed crops (Extended Data Fig. 2b and Supplementary Table 2). Conversely, having a single functional type beyond cereals enhanced only fats, while lowering calorie and other macronutrient outputs. Increased macronutrient outputs with crop diversity is in line with results from a global analysis^22^, which, however, did not explore the role of functional richness. Our study provides a comparison of macronutrient outputs of rotations with contrasting functional richness under the same management and pedoclimatic conditions. Given that increased functional richness brings gains to both calorie and macronutrient outputs, our results support the growing of crops in diverse rotations to safeguard food security without a need to expand agricultural land^18,23^. At the same time, we showed that the benefits occur only in functionally rich rotations and, in the case of carbohydrates, can be ascribed to monocultures yielding less and less, while functionally rich rotations provide unchanged or increasing outputs.

Several mechanisms can explain the enhanced calorie and macronutrient outputs at higher diversity. Functional richness can increase cereal crop yields as found in 32 long-term experiments across Europe and North America^10^, of which 15 were included here. Crop rotational diversity reduced cereal yield vulnerability to suboptimal growing conditions in several long-term experiments in Europe and North America^11,12,24,25^. Crop diversity also increased yields for non-cereal crops such as soybean^25,26^. These crops are more sensitive than cereals to the build-up of pests and pathogens when in monoculture, for example, root rot in legumes, club root in oilseed rape and nematodes in potatoes^27–29^. Moreover, biodiversity, soil fertility, pollination and pest control are enhanced by diverse rotations, which are likely to benefit all crops^4,30,31^.

**Extended Data Fig. 3:**
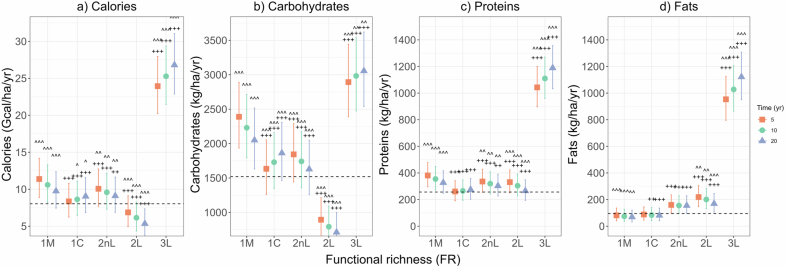
Model predicted effects of functional richness (FR) on annual crop rotation outputs when forage is used for milk (as Fig. 1 in the main text), but separating rotations with FR 2 into those including and not including ley (2L and 2nL respectively). Panels refer to calorie (**a**), carbohydrate (**b**), protein (**c**) and fat (**d**). All rotations with FR 3 include ley and are thus indicated as 3L. Forage crops are assumed to be used for milk production. The statistical models were obtained by fitting the 12,517 whole rotation outputs relative to the 16 long-term experiments (see Supplementary Table 1 for details). Filled symbols are model predictions for five (orange squares), ten (green diamonds), and twenty (blue triangles) years following the implementation of the rotation. Whiskers extend over the 5-95% confidence intervals. Symbols indicating significances refer to contrasts across levels of FR within each time, using FR 1 M at the same time as baseline ( + indicates *P*< 0.05; ++ *P* < 0.01; +++ *P*< 0.001) and across time within each FR level with time 0 at the same FR as baseline (^ indicates *P*< 0.05; ^^ *P*< 0.01; ^^^ *P*< 0.001). Post-hoc tests were two-sided and adjusted for multiplicity using multivariate t-distribution. Note the difference in the y axis scale among plots.

The difference in output between rotating two or three functional types was probably an effect of species and functional diversity, but not necessarily of the presence of ley. Ley is a perennial crop that strengthens multiple ecosystem functions such as soil quality and fertility, pest and weed regulation, and nitrogen use efficiency beyond crop diversity per se^32–35^. Nevertheless, rotations with two functional types including ley did not produce more calories and macronutrients than rotations with two functional types not including ley (Extended Data Fig. 3). In other words, ley did not appear to enhance outputs to a greater extent than the addition of another functional type to cereal rotations. Beyond strengthening ecosystem functions, ley contributes to rotation crude protein and metabolizable energy outputs^35^. However, these contributions alone did not compensate for the comparatively lower outputs from a ley crop in terms of calories and macronutrients per unit area, even when converted to milk (Supplementary Table 2).

Calorie and macronutrient outputs increased over time in rotations with three functional crop types, assuming that forage was used for milk production. This was in line with our third hypothesis and underlines the long-term benefits of diverse rotations. Interestingly, calorie and carbohydrate outputs also increased over time in cereal-only rotations, whereas outputs declined over time in cereal monocultures and in rotations with two functional types. Some of these trends were lost when removing data from the Padova site (Supplementary Fig. 3). Yield trends over time result from the combination of technological and variety improvements, changes in environmental conditions and crop rotational diversity. While some non-cereal crops, for example, legumes, are in social–technological lock-in^36^, staple cereals have been targeted for substantial genetic and agronomic improvements^37^. The decline in output over time for cereal monocultures suggests that crop variety and technological improvement do not compensate for detrimental effects from changing climatic conditions, soil degradation, and pest and weed build-up^4^. Rotating diverse cereals probably balanced some of these long-term effects, explaining the increase over time of the calorie and carbohydrate outputs of cereal-only rotations, despite their low functional richness. Differences in output trends between rotations with two or three functional types were probably an effect of species diversity, and their differing agronomic improvements^36,37^, responses to climate trends and susceptibility to pest build-up^27–29^, but they were not explained by the presence of ley (Extended Data Fig. 3). In summary, maximizing calorie and macronutrient outputs from diverse rotations requires careful consideration not only of functional richness, but also of the characteristics of each crop included in the rotation and their combined effects.

Calorie and macronutrient outputs for human consumption depended on the assumption of how forage crops were used. When converted into beef or biofuel, all outputs of functionally rich rotations were lower than those of cereal monocultures even after 20 years, except fats if forage was used for beef (Fig. 2 and Extended Data Tables 1 and 2). In other words, using arable crops to produce meat or biofuel causes large losses of human-available calories and macronutrients, whereas multiple benefits are achieved if forage is used for milk production. Note, however, that we did not account for the use of residues of food production for feed. This simplification penalized in particular carbohydrate and protein outputs of rotations that included oilseed crops. Moreover, the conversion coefficients from yield to calories and macronutrients (Supplementary Table 2) were assumed to be independent of location, rotational composition, position in rotation, time and other experimental treatments. This probably underestimated protein outputs at high functional richness, because cereal protein content has been shown to increase with legumes and oilseed crops in the rotation as their residues supply nitrogen to the following crop^38,39^. Cereal protein has also been shown to increase with water and heat stress^40–42^, and decrease with reduced soil nitrogen^40,41^ and enhanced atmospheric CO_2_ concentration^42^.

Functionally rich rotations could support healthier diets and provide local availability of these diets. Crop rotations including three functional types produced a distribution of macronutrients that is better aligned with current dietary recommendations, compared with cereal-only rotations. Guidelines for the USA recommend that the average adult obtains 45%–65% of calories from carbohydrates, 10%–35% from proteins and 20%–35% from fats^43^. Carbohydrates and proteins provide approximately 4 kcal kg^−1^ and fats 9 kcal kg^−1^ (ref. ^44^). On the basis of our results, the calories of a rotation including three functional types and with forage crops used to produce milk were 45% from carbohydrates, 18% from proteins and 39% from fats 20 years after implementation. By contrast, the calories from a cereal monoculture were 85% from carbohydrates, 11% from proteins and 8% from fats. A cereal-only rotation produced a similar balance of macronutrients. The macronutrient distribution of the diverse rotation using forage for milk was thus close to the recommended intake, whereas cereal-only rotations provided more carbohydrates and fewer fats than recommended. This dietary balance persisted also when outputs from the Padova experiment were not included. Hence, diverse rotations provide macronutrient proportions closer to a recommended human diet also when carbohydrate outputs are lower in the diverse rotation. If forage was used for beef or biofuel production, the diverse rotation produced an excess of carbohydrate and a shortage of fats, compared with the recommended intake, owing to the different nutritional composition and crop-to-retail ratio of milk and beef (Supplementary Table 2). Functionally rich rotations can thus support healthier diets than cereal-only cropping^45–47^, but the dietary advantage is lost if forage crops are used to produce meat or biofuel and non-food products. Diverse crop rotations have additional advantages, such as reducing synthetic fertilizer needs^9,10^ and the risk of yield losses through crop diversity insurance effects^48^, while improving farm economic performance^49^.

With an effective use of forage crops and crop residues, a higher crop diversity could support local access to more food and healthier diets compared with grain cropping systems. However, to increase the uptake of diverse rotations, changes are needed beyond the cropping system. Not all edible crops are currently used for direct human consumption, and the final use of many crops is affected by consumer preferences, including dietary choices, which are in turn influenced by public policies and food industry marketing. Moreover, the limited infrastructure for storage and transport, and the currently low market demands for alternative crops, create social–technological lock-ins that limit the adoption of diversified cropping systems^50,51^. Uptake of ley cropping would be facilitated by increased integration of grain and livestock farming in the landscape, which requires public and private policy to be redirected. Policies that support diversified cropping systems are needed to compensate for initial costs of investments and promote research and development of production, storage, processing and distribution^36^. Increasing crop diversity has potential to enhance food security and nutrition without agricultural land expansion.

## Methods

### Dataset

We assembled data from long-term field experiments across Europe, aiming at broad coverage of representative pedoclimatic conditions. We identified candidate long-term experiments via literature and referral from colleagues and contacted the reference person for each experiment for further information and access to data. A long-term experiment was retained based on five criteria. First, the experiments had to include a minimum of 10 years of yield data, although not necessarily consecutive. Second, the experiments had to comprise at least two rotations with contrasting numbers of crop species and/or rotation lengths, but not necessarily different FRs, each including at least a cereal. Consequently, if present, the continuous monoculture, that is, a single crop species cultivated every growing season, had to be a cereal. Moreover, all crops grown in the rotation had to be present every growing season and yield data had to be available for all crops. Last, any other treatment, such as tillage and fertilization, had to be fully crossed with the rotations or similar among the rotations, for example, pesticides applied as needed. Among the rotations established in each experiment, we retained only those including at least 1 year with a cereal crop in line with the selection criteria. When rotations were altered during the experiment, we restricted the analyses to the longest period with consistent management practice. In one site (Säby_LTE), we discarded the rotations with burned residues, considering only those with residues that were ploughed down.

The dataset resulted in 16 long-term experiments across Europe, differing in potential productivity (Extended Data Fig. 1), with 497 site-year combinations, 34,578 yield observations and 61 rotation-site combinations. Information on experiment location, duration, crop diversity and climatic conditions is summarized in Supplementary Table 1. The rotations established in each experiment had been deemed worth investigating by the local researchers and agricultural practitioners at each site. They can thereby be considered applicable in practical farming given the local pedoclimatic conditions, as well as societally relevant at least at the establishment of the experiment. Mono-sequences with the same cereal species represented 19% of the rotations. Leys, which are single or mixed plantings of perennial or biennial grasses and/or legumes, were included in 31% of the rotations. Ten per cent of the rotations comprised 1 year of fallow, providing no yield but allowing for the sowing of the following crop early. Mixtures of species occurred only in leys. No rotation had cover crops. In one of the long-term experiments, Tulloch, part of the ley was sheep grazed and neglected here, while the rest was used as silage and included in the analyses. The experimental design differed among sites, with some experiments including, for example, different levels of fertilizers, tillage and residue treatments. We combined these treatments with replicates into a grouping variable (hereafter ‘group’) to simultaneously account for the variation relative to replicate and treatment effects (Statistical Analyses). For example, two treatments (A and B) each replicated three times (1, 2 and 3) generated six groups: A1, A2, A3, B1, B2 and B3.

There were 2.28% missing yield data, and 0.92% of yields were reported as zero without any explanation. Explained zeroes include, for instance, fallow or annotated frost damage in winter crops. For each site and crop combination, the yield values that were below the first quartile defined for the crop and site minus twice the interquartile range or above the third quartile plus twice the interquartile range were checked for plausibility. Ultimately, based on expert opinion, we deemed 41 observations as unrealistically high, probably the result of incorrect reporting. Low but non-zero yields were kept because they probably reflect poor growing conditions. Missing and implausible values, as well as unexplained zeros, were gap filled by the average yield for the same crop, site, year, rotation and other treatments. The gap filling was impossible for 12 records, leading to the exclusion of the corresponding combination of site, rotation, year and other treatments from the output aggregation. Keeping the unmotivated zeros or not performing the gap filling did not alter the conclusions.

### Characterization of crop diversity

We quantified crop diversity based on functional richness (FR), that is, the number of functionally different crop types in the rotation. Ecological theories predict that functionally diverse plant communities have enhanced biomass production because plant functional types occupy different niches, thereby responding to abiotic variation differently, and drawing resources and suppressing the build-up of antagonists more efficiently^52^. We binned functionally similar crop species into four types: annual cereal, annual legume, annual broadleaf and ley, that is, biennial or perennial grass and/or legume in single or mixed plantings (Extended Data Fig. 2). This functional grouping, albeit coarse, has been shown to better explain variation in crop production compared with species richness^10,49^. Based on this, an FR of 1 corresponds to cereal-based systems, either as cereal monocultures (FR 1M, 57% of the rotations) or rotations with only cereal species (FR 1C). We considered FR 1M and FR 1C separately, because the greater diversity in a rotation with different cereal species could give higher outputs than a monoculture, or lower output if less productive cereals are combined. The maximum FR in our dataset was 3, with all rotations of this complexity including also ley (Extended Data Fig. 2). We categorized both root and oil crops as broadleaves, because only one rotation (Bd1 in Brody) included them both.

### Nutritional output of the entire rotation

We considered the nutritional output provided each year by all crops in each rotation, site, replicate and other treatments. To this aim, we summed all crop nutritional outputs produced in a single year to obtain total calorie and macronutrient (carbohydrate, protein and fat) outputs per cultivated area produced by the rotation in that growing season, thereby avoiding the confounding effects of interannual variability of growing conditions. We could do this because all experiments included all crops in the rotation each year. To make the outputs of rotations of different lengths comparable, we weighed them by the length of the rotation (as, for example, in refs. ^15,16^). This is equivalent to considering a 1-ha farm where a longer and more diverse rotation would entail smaller areas devoted to each crop during each year (Supplementary Fig. 4).

The nutritional value of each crop was obtained by converting the observed yield into total human-accessible calories and the macronutrient outputs of carbohydrates, proteins and fats for commonly used retail products. These were flour for cereals, raw beans for legumes, raw potatoes, swede or beets for root crops, seed oil for oil crops and sugar for sugar beet (Supplementary Table 2). The calories (Gcal ha^−1^ yr^−1^) or macronutrient (kg ha^−1^ yr^−1^) *n* provided by crop *c* in year *y* from experiment *j*, relative to the combination *k* of rotation and group, *M*_*n*,*c*,*y*,*j*,*k*_, is given by

1 $${M}_{n,c,y,j,k}={Y}_{c,y,j,k}{\left(1-{f}_{{{\rm{H}}}_{2}{\rm{O}},c}\right)}^{-1}{\alpha }_{c}\,{f}_{\mathrm{ref},c}{\gamma }_{n,c}.$$

Here *Y*_*c*,*y*,*j*,*k*_ is the dry yield of crop *c* per unit area (kg ha^−1^ yr^−1^). ${f}_{{{\rm{H}}}_{2}{\rm{O}},c}$ is the fraction of water in the retail product obtained from crop *c*. *α*_*c*_ is the crop-to-retail conversion factor, representing the amount of harvested yield necessary to obtain a unit mass of retail product (kg product per kg yield). *f*_ref,*c*_ is the refuse factor of the retail product, that is, the weight of the non-edible component per unit mass of retail product. γ_*n,c*_ is the nutrient conversion rate, accounting for the calories or macronutrient *n* contained in one unit mass of retail product of crop *c* (Gcal per kg product or kg per kg product, respectively; Supplementary Table 2). The water fraction, conversion and refuse factors are relative to the chosen product. All yields were transformed into dry weight based on their reported water content, if not already provided as dry weight.

The total output of calories or macronutrient *n* for year *y* in experiment *j* is the sum of the output contributions of all crops in the rotation weighed by the fraction of area under the *c*th crop, *F*_*A*,*c*,*k*_:

2 $${O}_{n,y,j,k}=\mathop{\sum }\limits_{c}{M}_{n,c,y,j,k}{F}_{A,c,k}.$$

*F*_*A*,*c*,*k*_ is calculated as 1/(rotation length).

We considered the calorie and macronutrient for direct human consumption when the harvested part of the crop was edible. However, ley, rye grass and forage rape crops serve only for forage. Other crops, such as maize, were grown for forage in some rotations and for direct human consumption in others (Supplementary Table 1). Forage crops can be used to produce dairy, meat, or biofuel and other non-food products. We compared three alternative conversion factors and their respective nutritional value for human consumption: forage for producing cow milk, boneless beef meat, and biofuel or other industrial products, that is, with no output for human nutrition. We assumed that 1.05 l of whole milk or 0.047 kg of boneless beef meat was produced per kg of dry matter intake of any forage crop (Supplementary Table 2), based on results from farms across the Netherlands^53^ and US Department of Agriculture^20^ respectively. These values are in line with several other estimates (Supplementary Table 2). In the Tulloch long-term experiment, we considered the outputs from the part of ley for silage, but not the sheep-grazed part, which was not quantified. This resulted in underestimated calorie and macronutrient outputs of these rotations.

The dataset comprised 12,517 whole-rotation outputs of calories or each macronutrient *n*. Of these, 35% referred to cereal mono-sequences with the same crop species (FR 1M); 18% were from rotations including ley, that is, single or mixed plantings of perennial or biennial grasses and/or legumes; and 6% were from rotations with 1 year of fallow (Extended Data Fig. 2).

### Statistical analyses

After aggregation, the whole-rotation outputs per hectare and year were analysed in four separate mixed-effects models, one for calories and one for each macronutrient. All models included FR and time as explanatory variables. A quadratic dependence on time since the implementation of the rotation was considered, to account for possible nonlinear changes in yields and hence calorie and macronutrient outputs over time, including stagnation or decline^10^. We also considered the interaction between time and FR, to account for the effects of crop diversity on yields over time (Supplementary Methods). Site was considered a random factor because the local pedoclimatic conditions affect yields (Extended Data Fig. 1) and sites can be interpreted as a sample from a larger population. We also had as a random factor the variable ‘group’ nested within site, to account for variation relative to experimental design, including replication and any treatment unrelated to functional richness. As a further random factor, we incorporated calendar year, to take into account local anomalous climatic conditions. Having calendar year as a random effect while also modelling time as a fixed effect allows us to consider both the likely trend in time, caused by technological improvements and changing climates, and the intrinsic year-to-year difference in growing conditions, which affect all rotations and crops in that site and year. In line with this reasoning, calendar year is a categorical variable and time is a continuous one. The dependent variable was root transformed to meet the assumption of normality of residuals. The fixed part of the model is as follows:

3 $$\sqrt{{O}_{n}}={\beta }_{0}+{\beta }_{t}t+{\beta}_{{t}^{2}}{t}^{2}+\mathop{\sum }\limits_{i}{\beta }_{{\mathrm{FR}}_{i}}{\mathrm{FR}}_{i}+\mathop{\sum}\limits_{i}{\beta}_{{\mathrm{FR}}_{i}t{\mathrm{FR}}_{i}t}+\mathop{\sum}\limits_{i}{\beta}_{{\mathrm{FR}}_{i}{t}^{2}}{\mathrm{FR}}_{i}{t}^{2}$$

where *O*_*n*_ is the total output of calories, carbohydrates, proteins or fats (subscript *n*), and *t* is time since the beginning of the experiment. *β*_0_ is the intercept, that is, the output of FR 1M when the rotations were first implemented. *β*_*t*_ and ${\beta}_{{t}^{2}}$ are the linear and quadratic effects of time. The summations extend over all the FR levels beyond monoculture so that the coefficients ${\beta }_{{\mathrm{FR}}_{i}}$, with *i* = 1C, 2, 3, are the contributions from FR 1C, 2 and 3 to the total output. ${\beta }_{{\mathrm{FR}}_{i}t}$ and ${\beta }_{{\mathrm{FR}}_{i}{t}^{2}}$ are the interaction effects between *t* and each FR_*i*_, and between *t*^2^ and each FR_*i*_, respectively.

The mixed-effects models were fitted using the ‘lmer’ function within the ‘lme4’ and ‘lmerTest’ packages (versions 1.1-37 and 3.1-3 respectively; refs. ^54,55^) in R, version 4.4.1. The assumptions of the models were checked by visual inspection of the residual plots using the ‘DHARMa’ package version 0.4.7 (ref. ^56^). Some minor deviations in tests emerged, as expected given the large sample size. However, residuals versus predicted values raised no concern; hence, no further action was taken. Predicted calorie and macronutrient outputs were calculated including the random component via the ‘ggeffects’ package version 1.7.0 (ref. ^57^). The contrasts were obtained via the ‘emmeans’, ‘contrast’ and ‘lsmeans’ functions of the ‘emmeans’ package version 1.11.1 (ref. ^58^) using the multivariate *t*-distribution. The R statements are reported in Supplementary Methods.

To assess the robustness of the results to the choice of long-term experiments, we removed one long-term experiment at a time and repeated the analyses, checking that the results would not be substantially affected by single experiments. The exclusion of long-term experiments Bologna or Broadbalk affected the significance of some factors and contrasts, although leading to similar conclusions (see details in Supplementary Results and Supplementary Figs. 1 and 2). Excluding the long-term experiment Padova cancelled the benefits of FR 3 to carbohydrates and some temporal trends (Supplementary Fig. 3), as discussed above. We also tested simpler random factor structures, specifically site alone, year nested in site and group nested in site, as well as their combinations, and found no appreciable changes in the conclusions but higher Akaike information criterion values compared with the structure described above.

### Reporting summary

Further information on research design is available in the Nature Portfolio Reporting Summary linked to this article.

## Supplementary information

Supplementary Information Supplementary Table 1, Supplementary Results, including Supplementary Figs. 1–3, and Supplementary Methods, comprising Supplementary Fig. 4, Supplementary Table 2 and R statements.

Reporting Summary

## Figures and Tables

**Extended Data Table 1:**
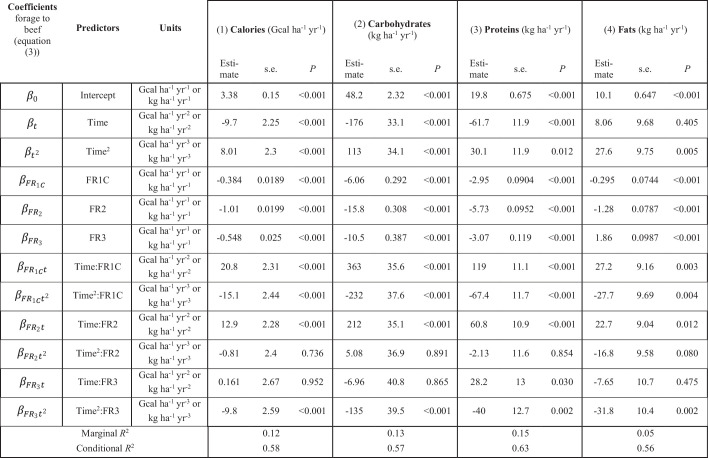
Effects of functional richness (FR) and time from implementation on annual crop rotation outputs (as Table 1 in the main text), but assuming forage crops are converted to beef

Fitted model parameters for the square-rooted annual outputs (equation (3)): (1) calories (Gcal ha^−1^ yr^−1^), (2) carbohydrates (kg ha^−1^ yr^−1^), (3) proteins (kg ha^−1^ yr^−1^), and (4) fats (kg ha^−1^ yr^−1^). The intercept is the output of a cereal monocultures, that is, the lowest level of FR (FR 1 M), at time 0. The two-sided *P* values were determined using the Satterthwaite’s approximation to calculate the degrees of freedom. The marginal *R*^2^ is the variation explained by the fixed factors alone, and the conditional *R*^2^ is the variation explained by the entire model including fixed and random factors.

**Extended Data Table 2:**
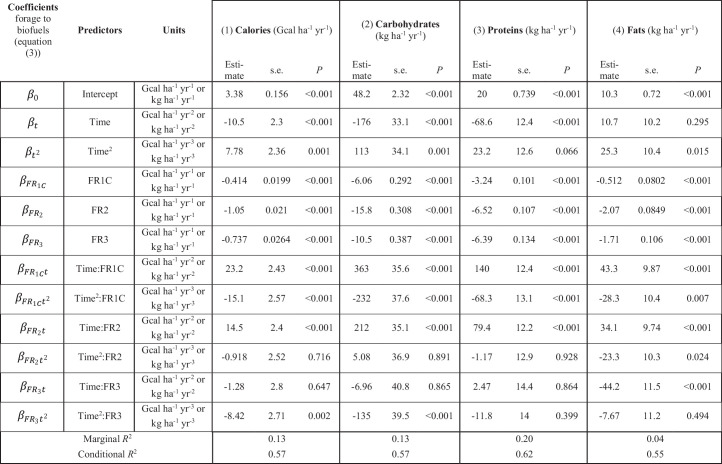
Effects of functional richness (FR) and time from implementation on annual crop rotation outputs (as Table 1 in the main text), but assuming forage crops are converted to biofuels

Fitted model parameters for the square-rooted annual outputs (equation (3)): (1) calories (Gcal ha^−1^ yr^−1^), (2) carbohydrates (kg ha^−1^ yr^−1^), (3) proteins (kg ha^−1^ yr^−1^), and (4) fats (kg ha^−1^ yr^−1^). The intercept is the output of a cereal monocultures (FR 1M) at year 0. The two-sided *P* values were determined using the Satterthwaite’s approximation to calculate the degrees of freedom. The marginal *R*^2^ is the variation explained by the fixed factors alone, and the conditional *R*^2^ is the variation explained by the entire model including fixed and random factors.

## Data Availability

Whole-rotation calorie and macronutrient outputs and metadata are available via the Swedish National Data Service, Researchdata.se (https://researchdata.se/en), at 10.5878/5q25-8572. Crop yield data are already available online for Broadbalk and can be made available upon reasonable request for the remaining sites.

## Code availability

The R statements relative to the model fitting, diagnostics and contrasts are reported in Supplementary Methods. The R code for the data analyses and metadata are available via the Swedish National Data Service, Researchdata.se (https://researchdata.se/en), at 10.5878/5q25-8572.
