# Supplementary material for: Functionally rich crop rotations increase calorie and macronutrient outputs across Europe
Source: Nat Food. 2026 Feb 25;7(2):185–93. doi: 10.1038/s43016-026-01293-5 (PMC12935540; doi:10.1038/s43016-026-01293-5)
Supplement: Supplementary file 1 — Supplementary Table 1, Supplementary Results, including Supplementary Figs. 1–3, and Supplementary Methods, comprising Supplementary Fig. 4, Supplementary Table 2 and R statements. [file 43016_2026_1293_MOESM1_ESM.pdf]

nature food

Supplementary information https://doi.org/10.1038/s43016-026-01293-5

# Functionally rich crop rotations increase calorie and macronutrient outputs across Europe

In the format provided by the authors and unedited

## Description of the Long-Term Experiments

**Table S1** Climatic conditions are summarized by annual mean temperature (T) and total precipitation (P), averaged over the years for which yield data are available, based on data from EObs[1] at 0.1° resolution v23.1e released in March 2021. Years of data indicate the period covered and the length of the record. Rotation length is the number of years needed to complete the rotation cycle. In crop rotation, crops followed by (f) are used as forage. Leys are specified as mixture of grasses only (grass), mixture of grasses and red clover (grass+rc), mixtures of grasses and white clover (grass+wc), and pure alfalfa (alfalfa). Cereals undersown with ley are denoted by *. Functional types in the rotations are cereals (C), broadleaves (BL), legumes (Leg) and ley (Ley). Fertiliser treatment indicates whether mineral (min), organic (org – manure or slurry) or a combination of both fertilisations (both) were used. In most sites, different fertiliser input levels were considered. Application rates of fertilisers and crop protection chemicals were either the same across diversity treatments or variable based on need. Tillage regimes (con: conventional; red: reduced) were always the same across diversity levels. Long-term experiment Tulloch was sheep grazed.

| Site (code), Country | Site location: Latitude, longitude | Climatic conditions T (°C), P (mm) | Years of data (length) | Rotation ID | Crop rotation | Crop species | Rotation length | FR | Functional types | Fertiliser Treatments (levels considered) | Tillage treatment | Crop protection | Study design | Number of replicates | Reference |
|---|---|---|---|---|---|---|---|---|---|---|---|---|---|---|---|
| Ås (As), Sweden | 63.25 °; 14.57 ° | T=3; P=553 | 1966: 2009 (44) | As1 | spring barley*, ley-grass+rc (f), ley-grass+rc (f), forage rape (f), potato, rye | 5 | 6 | 3 | C, BL, Ley | both, min (3) | conv | variable | split plot | 1 | [2] |
| | | | | As6 | spring barley | 1 | 1 | 1M | C | | | | | | |
| | | | | As7 | spring barley, spring barley, oat | 2 | 3 | 1C | C | | | | | | |
| | | | | As8 | spring barley, spring barley, potato | 2 | 3 | 2 | C, BL | | | | | | |
| | | | | As9 | spring barley*, ley-grass+rc (f), ley-grass+rc (f), | 2 | 3 | 2 | C, Ley | | | | | | |
| Bologna (Bo), Italy | 44.55 °; 11.35 ° | T=14; P=611 | 1984: 2020 (36) | Bo1 | maize, winter wheat, maize, winter wheat, maize, winter wheat, ley–alfalfa (f), ley-alfalfa (f), ley-alfalfa (f) | 3 | 9 | 2 | C, Ley | org, both, min (8) | conv | variable | split plot | 2 | [3] |
| | | | | Bo2 | maize, winter wheat | 2 | 2 | 1C | C | | | | | | |
| | | | | Bo4 | maize | 1 | 1 | 1M | C | | | | | | |
| | | | | Bo5 | winter wheat | 1 | 1 | 1M | C | | | | | | |
| Broadbalk (Bb), UK | 51.82 °; -0.35 ° | T=11; P=650 | 2000: 2017 (18) | Bb1 | winter wheat | 1 | 1 | 1M | C | org, min, both (17) | conv | same | split-split plot | 1 for Bb1, 5 for Bb2 | [4] |
| | | | | Bb2 | oats, maize(f), winter wheat, winter wheat, winter wheat | 3 | 5 | 1C | C | | | | | | |
| Brody (Bd), Poland | 52.43 °; 16.3 ° | T=9; P=548 | 1986: 1999 (14) | Bd3 | spring barley | 1 | 1 | 1M | C | min, both (1) | conv | same | split plot | 4 | [5,6] |
| | | | | Bd2 | winter rye | 1 | 1 | 1M | C | | | | | | |
| | | | | Bd1 | potato, spring barley, ley-alfalfa (f), ley-alfalfa (f), winter oilseed | 5 | 7 | 3 | C, BL, | | | | | | |

| | | | | | | | | | | | | | | | |
|---|---|---|---|---|---|---|---|---|---|---|---|---|---|---|---|
| | | | | | rape, winter rye, winter rye | | | | Ley | | | | | | |
| La Canaleja (LC), Spain | 40.35 °; -3.33 ° | T=14; P=396 | 2010: 2020 (11) | LC1 | winter wheat | 1 | 1 | 1M | C | min (1) | conv, red, none | variable | split plot | 4 | 7–9 |
| | | | | LC2 | winter wheat, vetch (f), winter barley, fallow | 4 | 4 | 2 | C, Leg | | | | | | |
| Lanna_ley (LL), Sweden | 58.33 °; 13.12 ° | T=7; P=612 | 1981: 2018 (38) | LL1 | winter oilseed rape, winter wheat, oats, spring barley*, ley-grass+rc (f), ley-grass+rc (f) | 6 | 6 | 3 | C, BL, Ley | min (4) | conv | variable | split plot | 1 | 10 |
| | | | | LL2 | winter oilseed rape, winter wheat, oats, spring barley*, ley-grass (f), ley-grass (f), | 6 | 6 | 3 | C, BL, Ley | | | | | | |
| | | | | LL3 | winter oilseed rape, winter wheat, oats, spring barley, spring wheat, fallow | 6 | 6 | 2 | C, BL | | | | | | |
| Öjebyn (Oj), Sweden | 65.35 °; 21.38 ° | T=2; P=547 | 1966: 2009 (44) | Oj1 | spring barley*, ley-grass+rc (f), ley-grass+rc (f), forage rape (f), potato, rye | 5 | 6 | 3 | C, BL, Ley | both, min (3) | conv | variable | split plot | 1 | 2 |
| | | | | Oj6 | spring barley | 1 | 1 | 1M | C | | | | | | |
| | | | | Oj7 | spring barley, spring barley, oats | 2 | 3 | 1C | C | | | | | | |
| | | | | Oj8 | spring barley, spring barley, potato | 2 | 3 | 2 | C, BL | | | | | | |
| | | | | Oj9 | spring barley*, ley-grass+rc (f), ley-grass+rc (f), | 2 | 3 | 2 | C, Ley | | | | | | |
| Osiny (Os), Poland | 51.46 °; 22.05 ° | T=9; P=555 | 1996: 2019 (24) | Os1 | winter wheat | 1 | 1 | 1M | C | min (1) | conv | variable | block design | 1 | 11 |
| | | | | Os2 | winter oilseed rape, winter wheat, spring barley (1996-2003)/spring wheat (2004-2019) | 3 | 3 | 2 | C, BL | | | | | | |
| Padova (Pa), Italy | 45.35 °; 11.97 ° | T=14; P=719 | 1990: 2019 (30) | Pa1 | winter wheat | 1 | 1 | 1M | C | both, min (2) | conv | variable | split plot | 3 | 12 |
| | | | | Pa2 | maize | 1 | 1 | 1M | C | | | | | | |
| | | | | Pa3 | winter wheat, maize | 2 | 2 | 1C | C | | | | | | |
| | | | | Pa4 | maize, sugar beet, maize, winter wheat, ley-alfalfa (f), ley-alfalfa (f) | 4 | 6 | 3 | C, BL, Ley | | | | | | |

| | | | | | | | | | | | | | | | |
|---|---|---|---|---|---|---|---|---|---|---|---|---|---|---|---|
| Perugia (Pe), Italy | 42.96 °; 12.38 ° | T=14; P=762 | 1974: 2013 (40) | Pe1 | winter wheat | 1 | 1 | 1M | C | | | | | | |
| | | | | Pe2 | winter wheat, maize | 2 | 2 | 1C | C | | | | | | |
| | | | | Pe3 | winter wheat, winter wheat, maize | 2 | 3 | 1C | C | | | | | | |
| | | | | Pe4 | winter wheat, winter wheat, winter wheat, maize | 2 | 4 | 1C | C | | | | | | |
| | | | | Pe5 | winter wheat, winter wheat, winter wheat, winter wheat, maize | 2 | 5 | 1C | C | | | | | | |
| | | | | Pe6 | winter wheat, winter wheat, winter wheat, winter wheat, winter wheat, maize | 2 | 6 | 1C | C | | | | | | |
| | | | | Pe8 | winter wheat, sunflower | 2 | 2 | 2 | C, BL | | | | | | |
| | | | | Pe9 | winter wheat, sorghum | 2 | 2 | 1C | C | | | | | | 13 |
| | | | | Pe10 | winter wheat, fava bean | 2 | 2 | 2 | C, Leg | min (1) | conv | variable | split plot | 3 | |
| Röbäcksdalen (Rb), Sweden | 63.82 °; 20.28 ° | T=3; P=636 | 1966: 2009 (44) | Rb1 | spring barley*, ley-grass+rc (f), ley-grass+rc (f), forage rape (f), potato, rye | 5 | 6 | 3 | C, BL, Ley | both, min (3) | conv | variable | split plot | 1 | 2 |
| | | | | Rb6 | spring barley | 1 | 1 | 1M | C | | | | | | |
| | | | | Rb7 | spring barley, spring barley, oats | 2 | 3 | 1C | C | | | | | | |
| | | | | Rb8 | spring barley, spring barley, potato | 2 | 3 | 2 | C, BL | | | | | | |
| | | | | Rb9 | spring barley*, ley-grass+rc (f), ley-grass+rc (f), | 2 | 3 | 2 | C, Ley | | | | | | |
| Säby_ley (SL), Sweden | 59.82 °; 17.70 ° | T=6; P=620 | 1981: 2016 (36) | SL1 | winter oilseed rape, winter wheat, oats, spring barley*, ley-grass+rc (f), ley-grass+rc (f) | 6 | 6 | 3 | C, BL, Ley | min (4) | conv | variable | split plot | 1 | 10 |
| | | | | SL2 | winter oilseed rape, winter wheat, oats, spring barley*, ley-grass (f), ley-grass (f) | 6 | 6 | 3 | C, BL, Ley | | | | | | |
| | | | | SL3 | winter oilseed rape, winter wheat, oats, spring barley, spring wheat, fallow | 6 | 6 | 2 | C, BL | | | | | | |

| | | | | | | | | | | | | | | | |
|---|---|---|---|---|---|---|---|---|---|---|---|---|---|---|---|
| Säby_LTE (SLTE), Sweden | 59.82 °; 17.70 ° | T=6; P=614 | 1974: 2010 (36) | SLTE4 | fallow, winter oilseed rape, winter wheat, oats, barley, spring wheat | 6 | 6 | 2 | C, BL | min (4) | conv | variable | split plot | 2 | [10] |
| | | | | SLTE5 | spring oats | 1 | 1 | 1M | C | | | | | | |
| | | | | SLTE6 | spring barley | 1 | 1 | 1M | C | | | | | | |
| | | | | SLTE7 | spring wheat | 1 | 1 | 1M | C | | | | | | |
| Stenstugu_ley (St), Sweden | 57.60 °; 18.43 ° | T=7; P=571 | 1968: 2020 (53) | St1 | winter oilseed rape, winter wheat, oats, spring barley*, ley-grass+rc (f), ley-grass+rc (f) | 6 | 6 | 3 | C, BL, Ley | min (4) | conv | variable | split plot | 1 | [10] |
| | | | | St2 | winter oilseed rape, winter wheat, oats, spring barley*, ley-grass (f), ley-grass (f) | 6 | 6 | 3 | C, BL, Ley | | | | | | |
| | | | | St3 | winter oilseed rape, winter wheat, oats, spring barley, spring wheat, fallow | 6 | 6 | 2 | C, BL | | | | | | |
| Tulloch (Tu), UK | 57.18 °; -2.25 ° | T=9; P=796 | 1991: 2006 (16) | TuE1 | ley-grass+wc (f), ley-grass+wc (f), ley-grass+wc (f), spring oats, swedes, oats* | 3 | 6 | 3 | C, BL, Ley | org (1) | conv | none | ran-dom-ised block | 2 | [14,15] |
| | | | | TuE2 | ley-grass+wc (f), ley-grass+wc (f), ley-grass+wc (f), ley-grass+wc (f), spring oats, oats* | 2 | 6 | 2 | C, Ley | | | | | | |
| Woodside (Ws), UK | 57.63 °; -3.40 ° | T=9; P=706 | 1991: 2012 (12) | Ws1 | ley-grass+wc (f), ley-grass+wc (f), ley-grass+wc (f), spring oats, potatoes, oats* | 3 | 6 | 3 | C, BL, Ley | org (1) | conv | none | ran-dom-ised block | 2 | [14,15] |
| | | | | Ws2 | ley-grass+wc (f), ley-grass+wc (f), spring oats, potatoes, oats*, ley-grass+wc (f), swedes, oats* | 4 | 8 | 3 | C, BL, Ley | | | | | | |

## Supplementary Results

To assess the effect of the selected long-term experiments and the robustness of our conclusions, we repeated the analyses by leaving out one long-term experiment at a time and checking differences in the sign of statistically significant (at $p<0.005$) coefficients. Removing data relative to the experiments in Bologna, Broadbalk or Padova influenced some of the contrasts, but not our main conclusions, as discussed next.

Removing long-term experiment Bologna resulted in larger reductions in carbohydrate outputs at FR 1C and smaller at FR 2, compared with the entire dataset, although both remained lower than FR 1M irrespective of time (Figure S1). Fat outputs at FR 1C were not different from those at FR 1M. Time had no longer an effect on calories at FR 1C till after 10 years since implementation and on fats at FR 1M 20 yrs after implementation.

Removing long-term experiment Broadbalk resulted in a benefit of FR 1C over 1M for carbohydrates (Figure S2), instead of a loss when considering the entire dataset. The fat benefits of rotating different cereals emerged already 5 years after implementation. Proteins at FR 1C were the same of FR 1M. Calories and carbohydrates remained stable over time at FR 1C, whereas a slight decline in fats emerged 10 years after the implementation.

After removing data from long-term experiment Padova, carbohydrates from FR 3 were lower than from FR 1M. Moreover, calories, carbohydrates and fats at FR 1M, as well as carbohydrates at FR 3, were no longer affected by time elapsed after rotation implementation, whereas a positive trend in proteins from FR 1C emerged. (Figure S3).

Long-term experiments Bologna and Broadbalk contrasted winter wheat (and maize) monocultures with a two- or five-year cereal only rotation, including maize and winter wheat (Table S1) and have high or intermediate potential productivity (Extended Data Figure 1).

This explains the positive changes in carbohydrates at FR 1C. Long-term experiment Padova comprised winter wheat and maize monocultures and a six-year FR 3 rotation, including two years of maize and one of sugar beet. This starch-rich rotation in a highly productive site (Extended Data Figure 1) likely affected the carbohydrate benefits at FR 3.

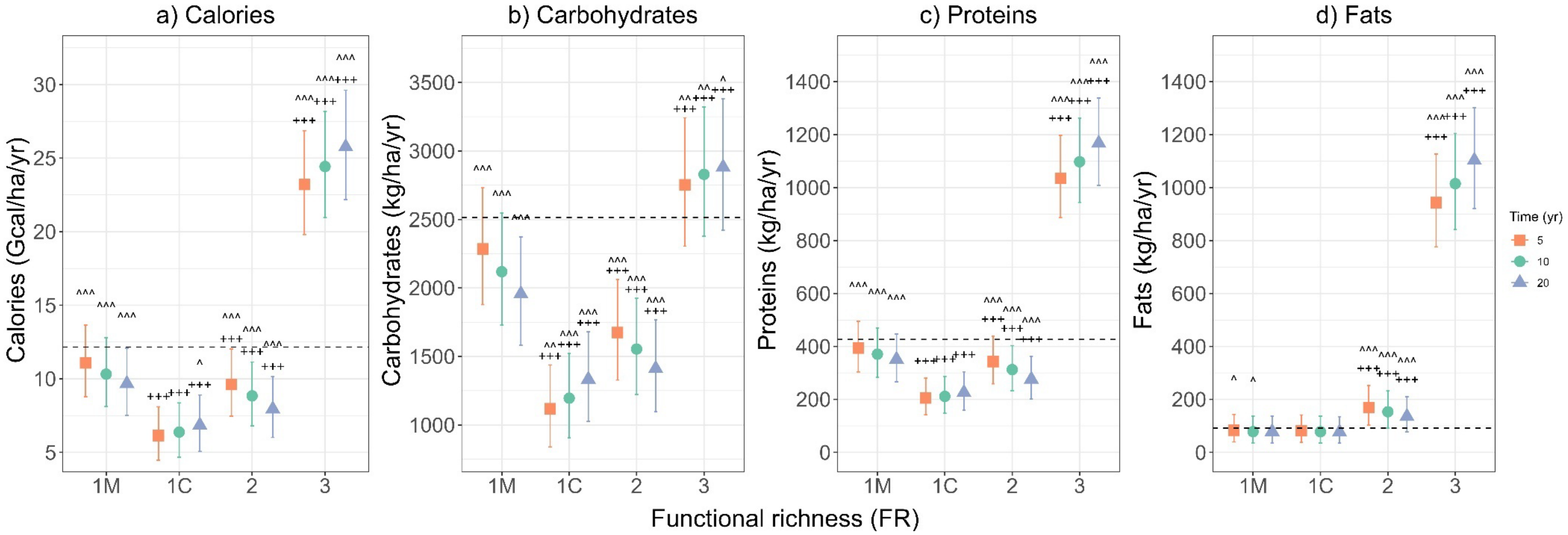


**Figure S1** Same as Figure 1 in the main text but excluding long-term experiment Bologna from the analyses. The statistical models are based on fitting the 10137 whole rotation outputs relative to the remaining 15 long-term experiments (see Table S1 for details). Forage crops were assumed to be used for milk production. Closed symbols are model predictions relative to five (orange squares), ten (green diamonds), and twenty (blue triangles) years following the implementation of the rotation. Whiskers extend over the 5 to 95% confidence intervals. Symbols indicating significances refer to contrasts across levels of FR within each time, using FR 1M at the same time as baseline (+ indicates $p<0.05$; ++ $p<0.01$; +++ $p<0.001$) and across time within each FR level with time 0 at the same FR as baseline (^ indicates $p<0.05$; ^^ $p<0.01$; ^^^ $p<0.001$). *Post-hoc* tests were two-sided and adjusted for multiplicity using multivariate t-distribution. Note the difference in the y axis scale among plots.

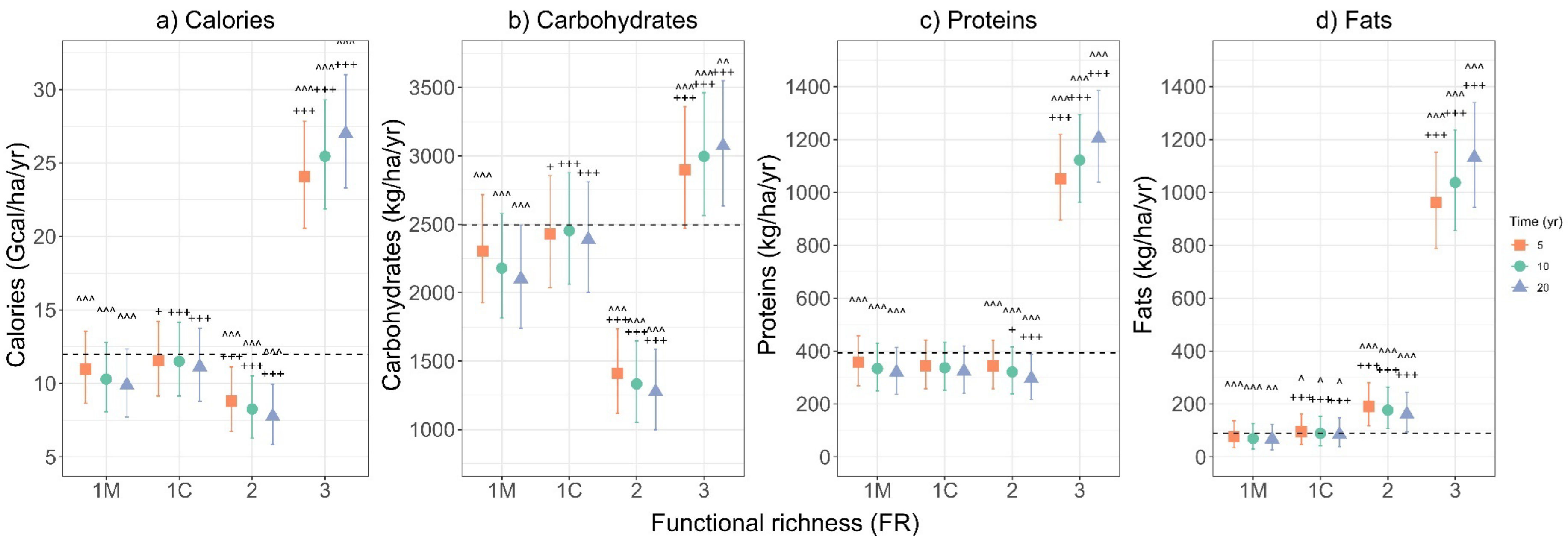


**Figure S2** Same as Figure 1 in the main text but excluding long-term experiment Broadbalk from the analyses. The statistical models are based on fitting the 11051 whole rotation outputs relative to the remaining 15 long-term experiments (see Table S1 for details). The meaning of bars and symbols are detailed in the caption to Figure S1.

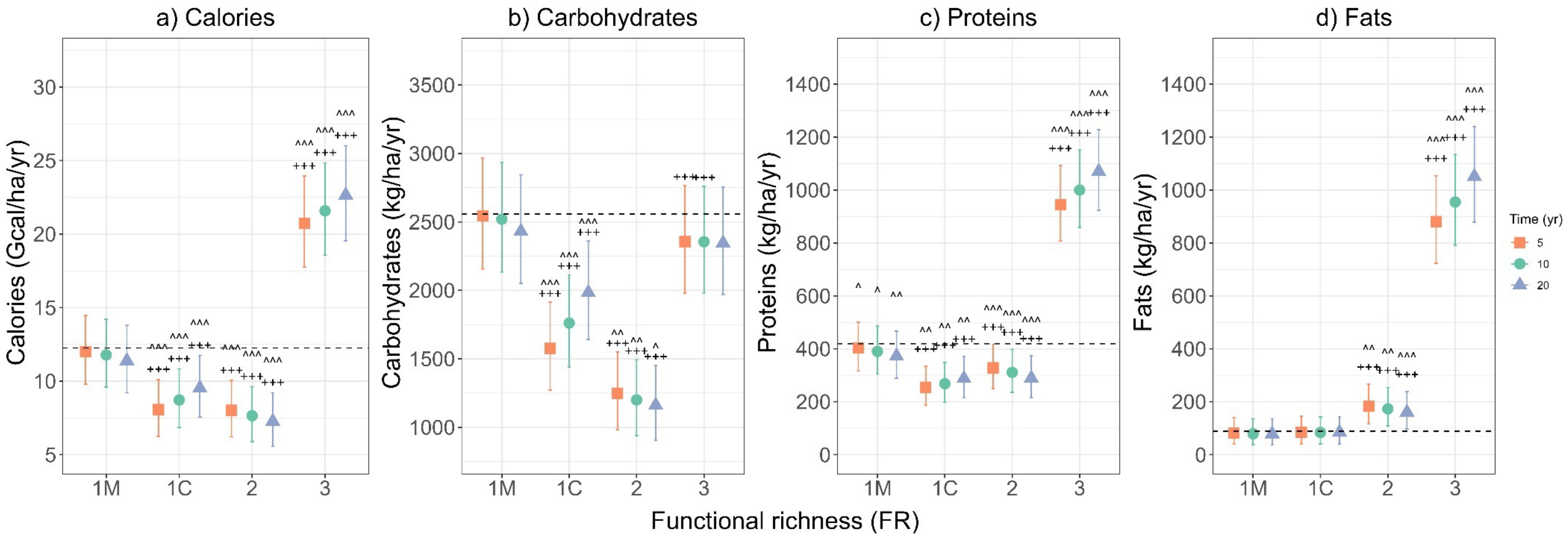


**Figure S3** Same as Figure 1 in the main text but excluding long-term experiment Padova from the analyses. The statistical models are based on fitting the 11865 whole rotation outputs relative to the remaining 15 long-term experiments (see Table S1 for details). The meaning of symbols is detailed in the caption to Figure S1.

## Supplementary Methods

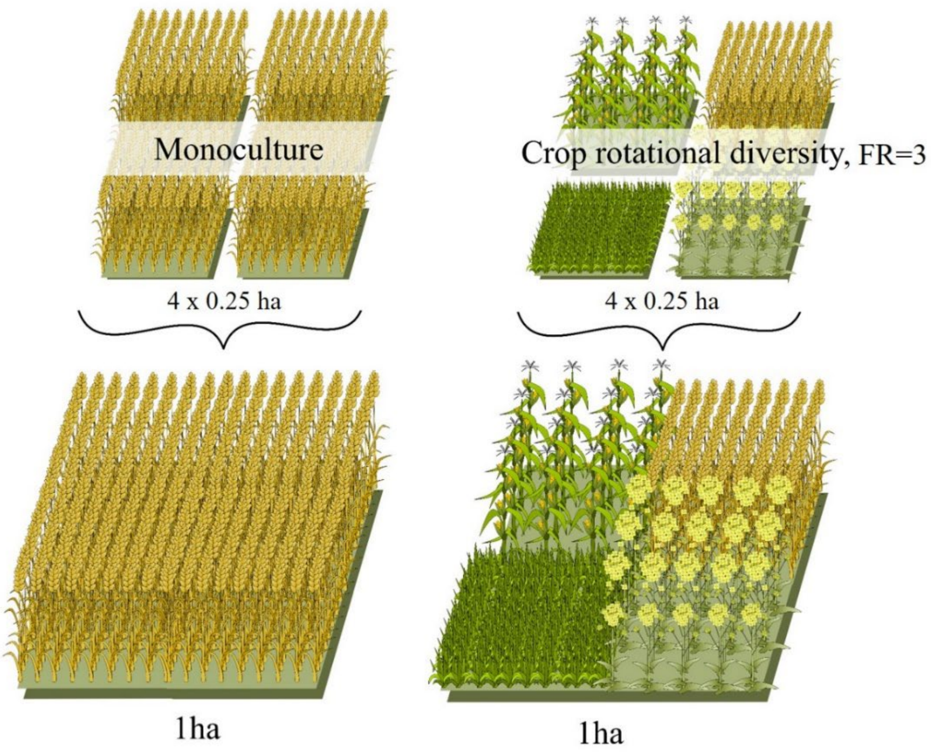


**Figure S4** Transformation of the amounts of calories or macronutrient produced by all crops in a year (per unit area) to total calorie or macronutrient output of the rotation during that year, assuming the rotation is implemented on a set area. The example considers a set area of 1 ha, either cultivated as cereal monoculture (i.e., the cereal is cultivated over the entire area; left) or as a rotation with FR 3, encompassing two cereals, one ley, and one oil crop (right). Since all crops are cultivated every year, each occupies a fraction of the set area proportional to the inverse of the rotation length, i.e., 1 ha for the cereal monoculture (left) and 0.25 ha for the four course rotation (right). Figure created by J Heinen.

**Table S2** Selected retail product, water, calorie and macronutrient contents, raw to retail conversion, and refuse factor relative to the crops included in the long-term experiments. Retail product is the abbreviated description of food items as per the USDA database (Shrt_Descr in https://www.ars.usda.gov/ARSUserFiles/80400525/Data/SR-Legacy/SR-Legacy_Doc.pdf; last accessed October 14th, 2025). Functional type refers to the classification of the crop. Crop to retail conversion factors for each crop $c$ and retail product, $\alpha_c$, were obtained from https://www.fao.org/fileadmin/templates/ess/documents/methodology/tcf.pdf. Columns denoted as calories, carbohydrates, proteins, and fats report the nutrient conversion rates, $\gamma_{n,c}$, for each nutritional output $n$ and crop $c$ (Eq. 1 in the Methods in the main text). $f_{H2O,c}$, $f_{ref,c}$, as well as calorie and macronutrient conversion rates $\gamma_{n,c}$ were sourced from https://www.ars.usda.gov/northeast-area/beltsville-md-bhnrc/beltsville-human-nutrition-research-center/food-surveys-research-group/docs/fndds-download-databases/, FNDDS 2019-2020, FNDDS Nutrient Values.xls and SR28 Dataset – table FOOD_DES (last accessed October 14th, 2025). The crop to retail conversion and water fraction for sugar beet was obtained from ref. [16], whereas the nutritional value of white sugar was obtained from the USDA as all the other products. The conversion factors for forage to milk and beef are based on results from farms across the Netherlands[17] and from USDA[18] respectively. These values are aligned with those emerging from other assessments throughout Europe (for milk, ref. [19–21]; for beef, ref. [20–22]), although other estimates are higher (for milk, ref. [22–24]; for beef, ref. [25,26]).

| **Crop** | **Retail product** | **Water fraction, $f_{H2O,c}$ (%)** | **Calories (kcal/100g)** | **Protein (%)** | **Fat (%)** | **Carbohydrate (%)** | **Raw crop to retail conversion, $\alpha_c$** | **Refuse factor, $f_{ref,c}$** |
|---|---|---|---|---|---|---|---|---|
| Barley | Barley, hulled | 9.44 | 354 | 12.48 | 2.3 | 73.48 | 0.72 | 0 |
| Broad beans dry | Broad beans (fava beans), mature seeds, raw | 10.98 | 341 | 26.12 | 1.53 | 58.29 | 1.0 | 0 |
| Fallow | Fallow | 0 | 0 | 0 | 0 | 0 | 0.0 | 1 |

| | | | | | | | | |
|---|---|---|---|---|---|---|---|---|
| Maize | Corn flour, whole grain | 10.91 | 361 | 6.93 | 3.86 | 76.85 | 0.82 | 0 |
| Oats | Oat flour, partly debranned | 8.55 | 404 | 14.66 | 9.12 | 65.7 | 0.53 | 0 |
| Oil seed rape | Oil, canola | 0 | 884 | 0 | 100 | 0 | 0.38 | 0 |
| Potatoes | Potatoes, flesh and skin, raw | 79.25 | 77 | 2.05 | 0.09 | 17.49 | 1.0 | 0.25 |
| Rutabaga/Swede | Rutabagas, raw | 89.43 | 37 | 1.08 | 0.16 | 8.62 | 1.0 | 0.15 |
| Rye | Rye flour, medium | 10.97 | 349 | 10.88 | 1.52 | 75.43 | 0.80 | 0 |
| Sorghum | Sorghum, flour, whole grain | 10.26 | 359 | 8.43 | 3.34 | 76.64 | 0.90 | 0 |
| Sugar beet | Sugar, white, granulated or lump | 76 | 401 | 0 | 0.32 | 99.6 | 0.16 | 0 |
| Sunflower seeds | Oil, sunflower, high oleic (>70%) | 0 | 884 | 0 | 100 | 0 | 0.41 | 0 |
| Wheat | Wheat flour, whole grain | 10.74 | 340 | 13.21 | 2.5 | 71.97 | 0.79 | 0 |
| *Products obtained from forage crops* | | | | | | | | |
| Cow milk, whole | Milk, whole, 3.25% fat, without added vitamin A and vitamin D | 88.13 | 61 | 3.15 | 3.27 | 4.78 | 1.05 | 0 |
| Beef, boneless | Beef, grass-fed, ground, raw | 67.13 | 198 | 19.42 | 12.73 | 0 | 0.047 | 0 |
| Biofuel | Biofuel | 0 | 0 | 0 | 0 | 0 | 1.0 | 0 |

**R statements**

We report below the R statements relative to the linear mixed model fitting, model diagnostic and contrasts used to generate the results. In these statements, output *O* is the whole rotation output (calories, carbohydrates, proteins or fats); the dataframe df contains the whole rotation outputs, as well as information on the site, year (time), group, functional richness (FR); back-transformed eff is used to plot the mean and confidence intervals; cntrFR and cntrt are the contrasts, represented in the plot with + and ^ symbols respectively. The code and a sample dataset are available from the Swedish National Data service, Researchdata.se (https://researchdata.se/en), doi: https://doi.org/10.5878/5q25-8572.

```
#linear mixed effect model fitting

FR_model=

lmerTest::lmer(sqrt(O)~poly(time,2)*FR+(1|site:year)+(1|site:group)+(1|site),data=df)

eff=ggeffects::ggeffect(FR_model,terms=c("FR","time[5,10, 20]"))


#model diagnostic

SO_FR= DHARMa::simulateResiduals(FR_model, n=200)


#contrasts

mod.rg <- emmeans::ref_grid(FR_model,at=list(FR=c("1M","1C","2","3"),time=c(0,5,10,20)))

emm <- emmeans::emmeans(regrid(mod.rg), ~ FR * time,
at=list(FR=c("1M","1C","2","3"),time=c(0,5,10,20)),type="response")

##contrasts within time, across FR

cntrFR= emmeans::contrast(emm, "trt.vs.ctrl", by = "FR",adjust="mvt")
```

##contrasts within FRs, across time

```
cntrt= emmeans::contrast(emm, "trt.vs.ctrl", by = "time",adjust="mvt")
```

**References**


1. Cornes, R. C., van der Schrier, G., van den Besselaar, E. J. M. & Jones, P. D. An Ensemble Version of the E-OBS Temperature and Precipitation Data Sets. *J. Geophys. Res. Atmospheres* **123**, 9391–9409 (2018).
2. Palmborg, C. *Soil and Crop Carbon and Nitrogen in an Experiment with Monocultures and Crop Rotations. A Study within the Project Climate CAFÉ.* 12 (2019).
3. Triberti, L., Nastri, A. & Baldoni, G. Long-term effects of crop rotation, manure and mineral fertilisation on carbon sequestration and soil fertility. *Eur. J. Agron.* **74**, 47–55 (2016).
4. Glendining, M. & Poulton, P. Dataset: Broadbalk Wheat annual grain and straw yields 1968-2022. Electronic Rothamsted Archive, Rothamsted Research https://doi.org/10.23637/rbk1-yld6822-01 (2023).
5. Blecharczyk, A., Kowalczewski, P. Ł., Sawinska, Z., Rybacki, P. & Radzikowska-Kujawska, D. Impact of Crop Sequence and Fertilization on Potato Yield in a Long-Term Study. *Plants* **12**, 495 (2023).
6. Blecharczyk, A., Sawinska, Z., Małecka, I., Sparks, T. H. & Tryjanowski, P. The phenology of winter rye in Poland: an analysis of long-term experimental data. *Int. J. Biometeorol.* **60**, 1341–1346 (2016).
7. Díaz-Ambrona, C. H. & Mínguez, M. I. Cereal–legume rotations in a Mediterranean environment: biomass and yield production. *Field Crops Res.* **70**, 139–151 (2001).
8. Soldevilla-Martinez, M. *et al.* Simulating improved combinations tillage-rotation under dryland conditions. *Span. J. Agric. Res.* **11**, 820–832 (2013).
9. Alonso, R. *et al. Legumbres - Salud Sostenible*. (Instituto Madrileño de Investigación y Desarrollo Rural, Agrario y Alimentario. IMIDRA, 2017).

10. Bergkvist, G. & Öborn, I. Long-term field experiments in Sweden - what are they designed to study and what could they be used for? *Asp. Appl. Biol.* 75–85 (2011).

11. Feledyn-Szewczyk, B., Jończyk, K. & Stalenga, J. The Effect of Crop Production Systems and Cultivars on Spring Wheat (Triticum aestivum L.) Yield in a Long-Term Experiment. *Agriculture* **14**, 625 (2024).

12. Morari, F., Lugato, E., Berti, A. & Giardini, L. Long-term effects of recommended management practices on soil carbon changes and sequestration in north-eastern Italy. *Soil Use Manag.* **22**, 71–81 (2006).

13. Bonciarelli, U. *et al.* Long-term evaluation of productivity, stability and sustainability for cropping systems in Mediterranean rainfed conditions. *Eur. J. Agron.* **77**, 146–155 (2016).

14. Taylor, B. R. *et al.* Output and sustainability of organic ley/arable crop rotations at two sites in northern Scotland. *J. Agric. Sci.* **144**, 435–447 (2006).

15. Watson, C. A. *et al.* Influence of ley duration on the yield and quality of the subsequent cereal crop (spring oats) in an organically managed long-term crop rotation experiment. *Org. Agric.* **1**, 147–159 (2011).

16. Babu, A. S. & Adeyeye, S. A. O. Chapter Seven - Extraction of sugar from sugar beets and cane sugar. in *Extraction Processes in the Food Industry* (eds Jafari, S. M. & Akhavan-Mahdavi, S.) 177–196 (Woodhead Publishing, 2024). doi:10.1016/B978-0-12-819516-1.00007-7.

17. van Boxmeer, E., Modernel, P. & Viets, T. Environmental and economic performance of Dutch dairy farms on peat soil. *Agric. Syst.* **193**, 103243 (2021).

18. Cassidy, E. S., West, P. C., Gerber, J. S. & Foley, J. A. Redefining agricultural yields: from tonnes to people nourished per hectare. *Env. Res Lett* **8**, 034015 (2013).

19. Wolf, P. *et al.* Assessing greenhouse gas emissions of milk production: which parameters are essential? *Int. J. Life Cycle Assess.* **22**, 441–455 (2017).

20. Röös, E., Patel, M. & Spångberg, J. Producing oat drink or cow's milk on a Swedish farm — Environmental impacts considering the service of grazing, the opportunity cost of land and the demand for beef and protein. *Agric. Syst.* **142**, 23–32 (2016).

21. Zira, S., Röös, E., Rydhmer, L. & Hoffmann, R. Sustainability assessment of economic, environmental and social impacts, feed-food competition and economic robustness of dairy and beef farming systems in South Western Europe. *Sustain. Prod. Consum.* **36**, 439–448 (2023).
22. Rundgren, G. *The Use of Feed for the Production of Meat, Egg and Cheese in Sweden*. 75 (2023).
23. Romano, E., Roma, R., Tidona, F., Giraffa, G. & Bragaglio, A. Dairy Farms and Life Cycle Assessment (LCA): The Allocation Criterion Useful to Estimate Undesirable Products. *Sustainability* **13**, 4354 (2021).
24. Gislon, G. *et al.* Forage systems and sustainability of milk production: Feed efficiency, environmental impacts and soil carbon stocks. *J. Clean. Prod.* **260**, 121012 (2020).
25. Nguyen, T. L. T., Hermansen, J. E. & Mogensen, L. Environmental consequences of different beef production systems in the EU. *J. Clean. Prod.* **18**, 756–766 (2010).
26. Mogensen, L. *et al.* Greenhouse gas emissions from beef production systems in Denmark and Sweden. *Livest. Sci.* **174**, 126–143 (2015).
