# Supplementary material for: Functionally rich crop rotations increase calorie and macronutrient outputs across Europe
Source: Nat Food. 2026 Feb 25;7(2):185–93. doi: 10.1038/s43016-026-01293-5 (PMC12935540; doi:10.1038/s43016-026-01293-5)
Supplement: Supplementary file 2 — Reporting Summary [file 43016_2026_1293_MOESM2_ESM.pdf]

nature portfolio

Corresponding author(s): Giulia Vico

Last updated by author(s): Nov 26, 2025

Reporting Summary

Nature Portfolio wishes to improve the reproducibility of the work that we publish. This form provides structure for consistency and transparency in reporting. For further information on Nature Portfolio policies, see our Editorial Policies and the Editorial Policy Checklist.

Statistics

For all statistical analyses, confirm that the following items are present in the figure legend, table legend, main text, or Methods section.

n/a	Confirmed	
☐	☒	The exact sample size (*n*) for each experimental group/condition, given as a discrete number and unit of measurement
☐	☒	A statement on whether measurements were taken from distinct samples or whether the same sample was measured repeatedly
☐	☒	The statistical test(s) used AND whether they are one- or two-sided<br>*Only common tests should be described solely by name; describe more complex techniques in the Methods section.*
☐	☒	A description of all covariates tested
☐	☒	A description of any assumptions or corrections, such as tests of normality and adjustment for multiple comparisons
☐	☒	A full description of the statistical parameters including central tendency (e.g. means) or other basic estimates (e.g. regression coefficient) AND variation (e.g. standard deviation) or associated estimates of uncertainty (e.g. confidence intervals)
☐	☒	For null hypothesis testing, the test statistic (e.g. *F*, *t*, *r*) with confidence intervals, effect sizes, degrees of freedom and *P* value noted<br>*Give P values as exact values whenever suitable.*
☒	☐	For Bayesian analysis, information on the choice of priors and Markov chain Monte Carlo settings
☐	☒	For hierarchical and complex designs, identification of the appropriate level for tests and full reporting of outcomes
☐	☒	Estimates of effect sizes (e.g. Cohen's *d*, Pearson's *r*), indicating how they were calculated

Our web collection on statistics for biologists contains articles on many of the points above.

Software and code

Policy information about availability of computer code

Data collection	No software was used
Data analysis	All data analyses were performed in R, version 4.4.1, using existing packaging, in particular lmert4 (v 1.1-37), lmerTest (v 3.1.3), DHARMa (v. 0.4.7), ggeffects (v. 1.7.0), emmeans (v. 1.11.1). The R statements for the analyses are reported in the Supplementary Information: Methods. The R code is available from the Swedish National Data service, Researchdata.se (https://researchdata.se/en), doi: https://doi.org/10.5878/5q25-8572

For manuscripts utilizing custom algorithms or software that are central to the research but not yet described in published literature, software must be made available to editors and reviewers. We strongly encourage code deposition in a community repository (e.g. GitHub). See the Nature Portfolio guidelines for submitting code & software for further information.

Data

Policy information about availability of data

All manuscripts must include a data availability statement. This statement should provide the following information, where applicable:

- Accession codes, unique identifiers, or web links for publicly available datasets
- A description of any restrictions on data availability
- For clinical datasets or third party data, please ensure that the statement adheres to our policy

Calorie and macronutrient outputs and metadata are made available through the Swedish National Data service, esearchdata.se (https://researchdata.se/en), doi: https://doi.org/10.5878/5q25-8572

Research involving human participants, their data, or biological material

Policy information about studies with human participants or human data. See also policy information about sex, gender (identity/presentation), and sexual orientation and race, ethnicity and racism.

Reporting on sex and gender	No human participants or human data are included
Reporting on race, ethnicity, or other socially relevant groupings	No human participants or human data are included
Population characteristics	No human participants or human data are included
Recruitment	No human participants or human data are included
Ethics oversight	N/A

Note that full information on the approval of the study protocol must also be provided in the manuscript.

Field-specific reporting

Please select the one below that is the best fit for your research. If you are not sure, read the appropriate sections before making your selection.

☐ Life sciences ☐ Behavioural & social sciences ☒ Ecological, evolutionary & environmental sciences

For a reference copy of the document with all sections, see nature.com/documents/nr-reporting-summary-flat.pdf

Ecological, evolutionary & environmental sciences study design

All studies must disclose on these points even when the disclosure is negative.

Study description	We contrasted whole rotation outputs of calories, carbohydrates, proteins and fats for human consumption relative to cereal monocultures, cereal-only rotations and rotations including also root or oil crops, legumes, and/or perennial ley. This study is based on yield data from 16 long-term experiments across Europe, for a total of 497 site-year combinations and 34578 yield observations relative to 61 crop rotation-site combinations, ranging from 1 to 4 functionally distinct crops. Long-term experiments differ in design structure and number of replicates (Supplementary Information, SI, Table S1). The yield data were transformed in calorie and macronutrient outputs based on selected retail products and the raw to retail conversion, refuse factor and calorie and macronutrient contents relative to each crops (SI, Table S2), assuming different uses of forage crops.
Research sample	The long-term experiments included in the study were chosen for representativity and broad coverage of pedoclimatic conditions within Europe. Candidate long-term experiments were identified via referral from colleagues and the literature, and the person(s) appearing as currently responsible for the experiment contacted. The long-term experiments included satisfy specific criteria (minimum of 10 years of data, at least two rotations, all rotations including cereals, all crops grown every year, and diversity not confounded with other treatments; see Methods for details). Whether a long term experiment met such criteria was confirmed with and the yield data were obtained from the person(s) currently responsible for of each long-term experiment. The person(s) currently responsible for the long-term experiments that met the above criteria were invited to contribute to this study and, as such, appear among the co-authors.
Sampling strategy	The study relies on the aggregation at the whole rotation of crop yield data previously collected within each long-term experiment.
Data collection	Yield data had been collected at each long-term field experiment, by personnel of each participating institution managing the long-term experiment and following the local protocol.
Timing and spatial scale	Data are available yearly for each replicated plot for 11 to 53 years depending on the long-term experiment. We included all the available data available in terms of years, rotations including cereals, and replicates, except when rotations were altered during the

	experiment. In this case, we selected the longest period with unchanged rotations. For one site (Säby_LTE), we did not consider the rotation with residue burning.
Data exclusions	No long-term experiment available to us and meeting the criteria detailed in the Methods were excluded. We considered unexplained zero yields as missing data and gap filled based on other replicates of the same crop grown in the same rotation during the same year. Considering the zeros as such does not alter the results.
Reproducibility	The robustness of results was tested by removing one long-term experiment at a time and verifying the main conclusions did not appreciably differ. Based on this approach, three long-term experiments were identified as changing some aspects of the results, although without altering our main conclusions. The results excluding those experiments are detailed in the SI.<br>Data of rotational outputs in terms of calories and macronutrients are available through the Swedish National Data service, Researchdata.se (https://researchdata.se/en), doi: https://doi.org/10.5878/5q25-8572
Randomization	In all long-term experiments, the rotations were set up in randomized designs. Plots were not replicated in 8 of the long-term experiments, but the length of the each record (11-53 years) allows for characterizing within-treatment variability.
Blinding	Blinding was not deemed necessary, because the yield data were collected following pre-defined local protocols aimed at standardization and to avoid researchers' bias.

Did the study involve field work? ☐ Yes ☒ No

Reporting for specific materials, systems and methods

We require information from authors about some types of materials, experimental systems and methods used in many studies. Here, indicate whether each material, system or method listed is relevant to your study. If you are not sure if a list item applies to your research, read the appropriate section before selecting a response.

Materials & experimental systems

n/a	Involved in the study	
☒	☐	Antibodies
☒	☐	Eukaryotic cell lines
☒	☐	Palaeontology and archaeology
☒	☐	Animals and other organisms
☒	☐	Clinical data
☒	☐	Dual use research of concern
☐	☒	Plants

Methods

n/a	Involved in the study	
☒	☐	ChIP-seq
☒	☐	Flow cytometry
☒	☐	MRI-based neuroimaging

Dual use research of concern

Policy information about dual use research of concern

Hazards

Could the accidental, deliberate or reckless misuse of agents or technologies generated in the work, or the application of information presented in the manuscript, pose a threat to:

No	Yes	
☒	☐	Public health
☒	☐	National security
☒	☐	Crops and/or livestock
☒	☐	Ecosystems
☒	☐	Any other significant area

Experiments of concern

Does the work involve any of these experiments of concern:

No	Yes	
☒	☐	Demonstrate how to render a vaccine ineffective
☒	☐	Confer resistance to therapeutically useful antibiotics or antiviral agents
☒	☐	Enhance the virulence of a pathogen or render a nonpathogen virulent
☒	☐	Increase transmissibility of a pathogen
☒	☐	Alter the host range of a pathogen
☒	☐	Enable evasion of diagnostic/detection modalities
☒	☐	Enable the weaponization of a biological agent or toxin
☒	☐	Any other potentially harmful combination of experiments and agents

Plants

Seed stocks	N/A
Novel plant genotypes	No novel plant genotypes produced
Authentication	N/A
